# Crystallization and first data collection of the putative transfer protein TraN from the Gram-positive conjugative plasmid pIP501

**DOI:** 10.1107/S174430911204184X

**Published:** 2012-10-30

**Authors:** Nikolaus Goessweiner-Mohr, Christian Fercher, Mohammad Yaser Abajy, Elisabeth Grohmann, Walter Keller

**Affiliations:** a Institute for Molecular Biosciences, Karl-Franzens-University Graz, Humboldtstrasse 50/III, 8010 Graz, Austria; b Environmental Microbiology/Genetics, Technical University Berlin, Franklinstrasse 28/29, 10587 Berlin, Germany; c Division of Infectious Diseases, University Medical Center Freiburg, Hugstetter Strasse 55, 79106 Freiburg, Germany

**Keywords:** Gram-positive, conjugative plasmid transfer, pIP501

## Abstract

Conjugative plasmid transfer is the most important route for the spread of resistance and virulence genes among bacteria. Consequently, bacteria carrying conjugative plasmids are a substantial threat to human health, especially hospitalized patients. Whilst detailed information about the process has been obtained for Gram-negative type-4 secretion systems, little is known about the corresponding mechanisms in Gram-positive (G+) bacteria. The successful purification and crystallization of the putative transfer protein TraN from the G+ conjugative model plasmid pIP501 of *Enterococcus faecalis* are presented. Native crystals diffracted to 1.8 Å resolution on a synchrotron beamline. The crystals belonged to space group *P*2_1_, with unit-cell parameters *a* = 32.88, *b* = 54.94, *c* = 57.71 Å, β = 91.89° and two molecules per asymmetric unit.

## Abstract 2

The successful purification, crystallization and first data collection to 1.8 Å resolution of the putative transfer protein TraN from the Gram-positive conjugative plasmid pIP501 are reported.

## 1. Introduction

Besides transformation and transduction, bacterial conjugation is the major mechanism of horizontal gene transfer. It is the prevalent means by which plasmid-encoded antibiotic resistance and toxicity genes are spread (Williams & Hergenrother, 2008 ▶). During conjugation, plasmid DNA is transported from a donor to a recipient cell by a multi-protein complex large enough to span the bacterial cell wall (Llosa *et al.*, 2002 ▶). Gram-negative (G−) bacteria with conjugative plasmids (*e.g. Escherichia coli* or *Agrobacterium tumefaciens*) make use of type-4 secretion (T4S) systems, multi-protein complexes which are dedicated to the intercellular transport of proteins or protein–DNA complexes (Smillie *et al.*, 2010 ▶; Wallden *et al.*, 2010 ▶; Thanassi *et al.*, 2012 ▶; Zechner *et al.*, 2012 ▶). The conjugation process generally requires cell-to-cell contact of the donor cell with the recipient cell to translocate substrates across the cell envelopes (Cascales & Christie, 2003 ▶; Alvarez-Martinez & Christie, 2009 ▶). Much more information regarding the individual function, regulation and interaction of the proteins involved in the T4S processes is available for G− bacteria (Grohmann *et al.*, 2003 ▶; Kurenbach *et al.*, 2006 ▶; Wallden *et al.*, 2010 ▶; Clewell, 2011 ▶). Furthermore, most knowledge about Gram-positive (G+) systems is based on homology to their G− counterparts (Grohmann *et al.*, 2003 ▶; Abajy *et al.*, 2007 ▶). However, some structural information on G+ transfer proteins has recently become available (Porter *et al.*, 2012 ▶; Walldén *et al.*, 2012 ▶). 

The multiple antibiotic-resistance plasmid pIP501 was originally isolated from *Streptococcus agalactiae* (Horodniceanu *et al.*, 1979 ▶). It has the broadest known host range for plasmid transfer in G+ bacteria and is the first self-transmissible plasmid from G+ bacteria for which stable replication has been demonstrated in G− bacteria (Kurenbach *et al.*, 2003 ▶). The transfer region of pIP501 is organized as an operon encoding 15 putative transfer (Tra) proteins. Three of these Tra proteins show significant sequence similarity to the T4S system from *A. tumefaciens*: an ATPase (TraE, a homologue of VirB4; Abajy *et al.*, 2007 ▶), a coupling protein (TraJ, a homologue of VirD4; E. K. Celik, W. Keller & E. Grohmann, unpublished work) and a lytic transglycosylase (TraG, a homologue of VirB1; K. Arends, W. Keller & E. Grohmann, unpublished work). In addition, TraA, the relaxase of the pIP501-encoded T4S system, has been functionally characterized (Kopec *et al.*, 2005 ▶; Kurenbach *et al.*, 2006 ▶).

Here, we present the purification and crystallization of TraN, a 17.6 kDa protein (formerly ORF14, GenBank CAD44394.1) of the transfer operon from the G+ conjugative plasmid pIP501. TraN is the second potential transfer protein of this system to be crystallized. A sequence-similarity search within G+ and G− T4S systems has not revealed any related proteins. Previously performed electrophoretic mobility shift assays (EMSAs; data not shown) mark TraN as a dsDNA-binding protein. Its possible functions include a role as an accessory protein for the pIP501-encoded relaxase TraA or as a transcription regulator adjusting the frequency of expression of key pIP501 transfer proteins. The high-resolution structure will be key to elucidating the function of TraN.

## 2. Protein purification

**Figure 1:**
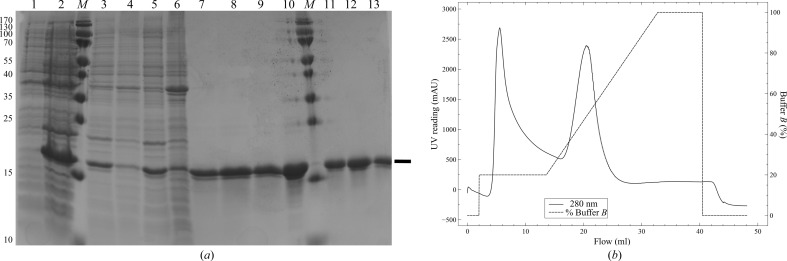
TraN protein production. (*a*) 18% SDS–polyacrylamide gel to assess protein production and purification (TraN, 17.6 kDa). Lanes 1 and 2, expression before and after 3 h IPTG induction; lanes 3 and 5, supernatant of the two-step extraction; lanes 4 and 6, pellet of the two-step extraction; lanes 7–9, main fractions of the His-affinity purification; lane 10, pooled and concentrated His-affinity fractions; lanes 11–13, main size-exclusion chromatography fractions; lanes *M*, molecular-mass marker (PageRuler SM0671, Thermo Fisher Scientific, Waltham, Massachusetts, USA; labelled in kDa). (*b*) His-affinity purification of TraN; the imidazole gradient is shown as the percentage of buffer *B* (discontinuous line).

*traN* was cloned into the 7×His-tag expression vector pQTEV (a gift from K. Büssow, Max-Planck-Institute for Molecular Genetics, Berlin, Germany), and *E. coli* BL21-CodonPlus (DE3)-RIL (Stratagene, Amsterdam, The Netherlands) competent cells were transformed with the recombinant construct pQTEV-*traN*. Large-scale expression was performed in 500 ml LB medium supplemented with 100 µg ml^−1^ ampicillin. TraN expression was induced at an OD_600_ of ∼0.6 by addition of 1 m*M* IPTG and expression continued for 3 h at 310 K. Cells were harvested and immediately frozen at 253 K. TraN expression levels were monitored by SDS–PAGE (Fig. 1 ▶ *a*).

TraN cell pellets were first lysed in 40 ml 25 m*M* HEPES pH 7.6, 75 m*M* Na_2_SO_4_ (buffer *A*). 2 U DNAse (Sigma–Aldrich, St Louis, USA), phenylmethanesulfonylfluoride and benzamidine (final concentrations of 1 and 2 m*M*, respectively) were added. The cell suspension was vigorously mixed (UltraTurrax, IKA, Staufen, Germany) and kept on ice for 30 min. The solution was sonicated (Sonopuls HD2070, Bandelin; 1 min, continuous sonication, ∼80% amplitude) and centrifuged for 30 min at 281 K and 15 000*g*. Pellet and supernatant fractions were analysed by SDS–PAGE. The pellet was applied to a second extraction step with 20 ml buffer *A*. The TraN-containing supernatant fractions were pooled and loaded onto a HisTrap FF 1 ml column (GE Healthcare, Chalfont St Giles, England) for affinity purification (Fig. 1 ▶ *b*). The purity of TraN was assessed by SDS–PAGE (Fig. 1 ▶ *a*). Imidazole was removed by buffer exchange during concentration (Amicon tubes, 3000 MWCO, Merck, Darmstadt, Germany).

**Figure 2:**
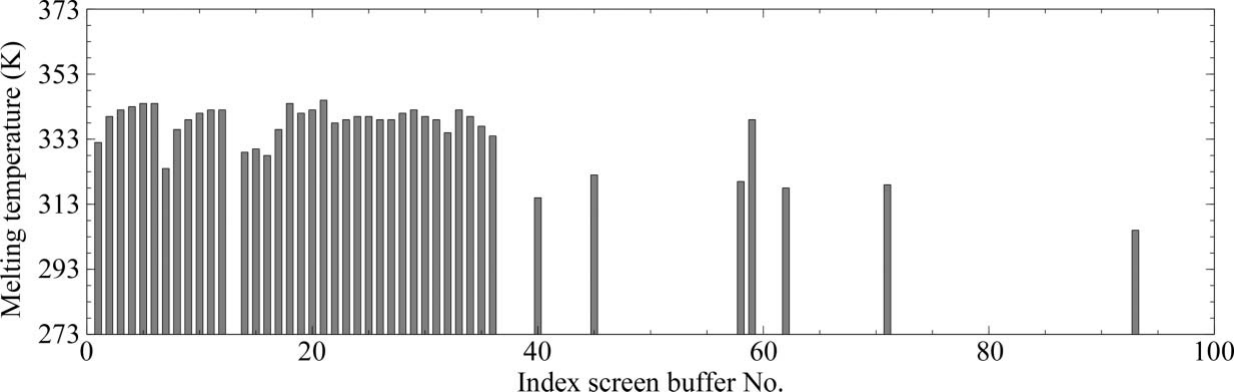
Example of the buffer-optimization assays. The melting temperatures (K) of TraN are plotted as a function of the buffer and differ significantly corresponding to the respective chemical composition. The values on the *x* axis correspond to the numbering of the Index crystallization screen; missing values represent melting curves that were measured but were not interpretable, which are likely to arise from precipitation or aggregation.

Purified TraN protein at a concentration of 1 mg ml^−1^ was subjected to an adapted differential scanning fluorometry buffer optimization screening (Ericsson *et al.*, 2006 ▶) using all crystallization buffers present in the Index, PEG/Ion, MembFac (Hampton Research, Aliso Viejo, California, USA) and Morpheus (Molecular Dimensions, Newmarket, England) screens (Fig. 2 ▶). For the assay, 10 µl protein sample was mixed with 10 µl of the respective buffer and 5 µl of a 50× SYPRO Orange (Sigma–Aldrich, St Louis, USA) stock. The resulting thermostability curves were analysed and a new extraction buffer was designed combining the buffer components (Collins *et al.*, 2004 ▶) which showed a thermostabilizing effect, while keeping the composition as simple as possible (TraN_lysis). This buffer consisted of 50 m*M* HEPES pH 7.6, 100 m*M* ammonium sulfate and was used for all subsequent TraN extractions, as well as for crystallization.

## 3. Crystallization

His-tagged TraN was initially set up with an Index screen at a stock concentration of 4.8 mg ml^−1^ using the microbatch method (Chayen *et al.*, 1992 ▶). After evaluation of this first plate, the following screens were prepared at different concentrations: Index, Crystal Screen, Crystal Screen 2, MembFac, PEG/Ion (Hampton Research), JCSG and Morpheus (Molecular Dimensions). The drop ratio was 1:1, with a total drop volume of 1 µl. All plates were covered with paraffin oil (∼4 ml in total) and stored at 293 K. The formation of crystals was monitored over several weeks. Potential protein crystals were tested for diffraction using a rotating-anode diffractometer (MicroStar, Bruker AXS, Madison, Wisconsin, USA). From several positive conditions, the two most promising, Index No. 42 [0.1 *M* bis-tris pH 5.5, 25%(*w*/*v*) PEG 3350] and No. 72 [0.2 *M* NaCl, 0.1 *M* HEPES pH 7.5, 25%(*w*/*v*) PEG 3350], were selected for microbatch pH/PEG/protein concentration optimization matrices. A constant protein drop volume of 1 µl and different protein stock concentrations were used.

**Figure 3:**
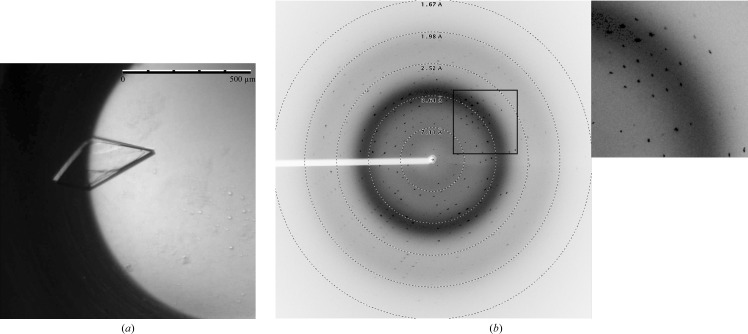
TraN crystallization and data collection. (*a*) A representative TraN crystal which grew very compactly to a maximal size of about 400 µm. The crystal was grown using the microbatch method at 293 K and using paraffin oil only to seal the plate. The protein drop ratio was 50% with a protein stock concentration of 4.6 mg ml^−1^. The drop size was 1 µl with the crystallization buffer Index condition No. 72: 0.2 *M* NaCl, 0.1 *M* HEPES pH 7.5, 25%(*w*/*v*) PEG 3350. (*b*) Diffraction pattern of a native TraN crystal obtained using synchrotron radiation on beamline X06DA, SLS, Villigen, Switzerland; resolution rings have been added. The picture was generated using *ADXV* (A. Arvai). Inset, detail of the diffraction shown in (*b*).

The original conditions showed thin and fragile platelets and the diffraction of these crystals was very anisotropic. The optimization did not improve the crystal diffraction limit or quality, but the use of the enhanced extraction buffer TraN_lysis led to the formation of thicker crystals with improved diffraction behaviour (see Fig. 3 ▶ for details of the setup). These optimized crystals were used for data collection at the synchrotron. We analysed dissolved crystals *via* matrix-assisted laser desorption/ionization–time of flight (MALDI–TOF) mass spectrometry (MS) measurements (ultrafleXtreme, Bruker, Vienna, Austria) to confirm the integrity of TraN in the crystals. The crystals were dissolved in 10 µl pure H_2_O. The MS analysis showed that TraN was significantly smaller than the original His-tagged construct. Two peaks of equal height were visible with molecular masses of 14 222 and 14 478 Da (data not shown), implying two cleavage sites with a difference of two amino acids. The His tag, including the TEV cleavage site and linker, amounts to a molecular weight of 3213 Da (ExPASy; Gasteiger *et al.*, 2003 ▶). This corresponds well to the difference between the full-length protein (17 566 Da; ExPASy) and the two species of TraN observed in the crystals. The measured masses correspond to calculated fragments within 20 Da. However, we cannot present the exact cleavage site because of the absence of N-terminal sequencing. We conclude that the N-terminal tag is cleaved by *in situ* proteolysis during the crystallization process.

## 4. Data collection and processing

Crystals were flash-cooled without cryoprotectant. Data collection was performed at 100 K on the synchrotron beamline X06DA at SLS, Villigen, Switzerland. The tested crystals diffracted to a resolution of about 3 Å on our home source and to 1.8 Å at the SLS. All tested crystals still showed severe anisotropic behaviour, diffracting to approximately 1.8 Å resolution in the best and 2.6 Å in the worst direction. A crystal with the most uniform diffraction pattern was chosen for a 180° data collection (Fig. 3 ▶) using a crystal-to-detector distance of 150 mm, an oscillation range of 1.0° and an exposure time of 1 s per image. Data-collection and processing statistics are given in Table 1 ▶.

The crystal used for the data collection belonged to space group *P*2_1_, with unit-cell parameters *a* = 32.88, *b* = 54.94, *c* = 57.71 Å, β = 91.89° and two molecules per asymmetric unit. The Matthews coefficient (Kantardjieff & Rupp, 2003 ▶) was calculated to be 1.78 Å^3^ Da^−1^, with a solvent content of 31.18% and a probability of 0.7 of being the most likely solution for the given resolution (Table 2 ▶). The data set was processed with *iMOSFLM* (Battye *et al.*, 2011 ▶) and scaled with *SCALA* (Evans, 2006 ▶) within the *CCP*4 software suite (Winn *et al.*, 2011 ▶). The *R*_meas_ of the scaled data was found to be relatively high (14.1%), likely owing to the inherent anisotropy of the data.

A self-rotation function was calculated with *MOLREP* (Vagin & Teplyakov, 2010 ▶) and analysed, showing no evidence of a second molecule. We also generated a native Patterson map with the *CCP*4 program *PEAKMAX* and found a peak at position *x* = 0, *y* = 0.0565, *z* = 0.5 with 40.2% intensity of the origin peak, indicating a second molecule related to the first molecule by pseudo-translation.

Since no known structures with sequence similarity are available, the TraN structure cannot be solved by molecular-replacement methods. Therefore, we are currently pursuing the structure solution of TraN by conventional heavy-atom-derivative methods. In parallel, we are in the process of expressing a selenomethionine derivative of TraN for crystallization and single-wavelength anomalous dispersion/multiple-wavelength anomalous dispersion (SAD/MAD) experiments. The native data presented here will be used in the refinement of a preliminary model obtained from these efforts.

## Figures and Tables

**Table 1:** Data-collection and processing statistics for the scaled data Values in parentheses are for the highest resolution shell.

Beamline	X06DA [PXIII], SLS, Villigen, Switzerland
Space group	*P*2_1_
Detector	MAR CCD
Unit-cell parameters (Å, °)	*a* = 32.88, *b* = 54.94, *c* = 57.71, β = 91.89
Wavelength (Å)	0.9794
Resolution range (Å)	28.967–1.8 (1.9–1.8)
*R*_meas_ † (%)	14.1 (61.6)
〈*I*/σ(*I*)〉	8.8 (3.7)
No. of molecules in asymmetric unit	2
Matthews coefficient (Å^3^ Da^−1^)	1.78
Solvent content (%)	31.18
Measured reflections	69059 (10004)
Unique reflections	19191 (2804)
Multiplicity	3.6 (3.6)
Completeness (%)	100.0 (100.0)

† *R*_meas_ = 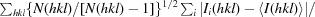 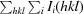.

**Table 2:** Results of the Matthews coefficient calculation Values were calculated for a molecular weight of 14 600 Da.

No. of molecules in asymmetric unit	Matthews coefficient (Å^3^ Da^−1^)	Solvent content (%)	Probability (*N*) for given resolution (1.8 Å)	Probability (*N*) overall
1	3.57	65.55	0.30	0.73
2	1.78	31.10	0.70	0.27
